# A Large Irritation Fibroma of the Hard Palate: A Rare Clinical Entity

**DOI:** 10.7759/cureus.62244

**Published:** 2024-06-12

**Authors:** Abhinav Srivastava, Pooja Bhati, Vishwani Khanna, Kanika Arora, Rohit Saxena

**Affiliations:** 1 Department of Otorhinolaryngology, School of Medical Sciences and Research, Sharda Hospital, Greater Noida, IND

**Keywords:** reactive hyperplasia, benign tumor, hard palate, fibroma, oral cavity

## Abstract

An irritative fibroma of the oral cavity can be defined as a benign tumor of connective tissue. They usually occur in the oral cavity, with the most common sites being the buccal mucosa and tongue. However, reported cases over the hard palate are few. Irritant or reactive fibromas are brought upon by recurrent, mildly intense stimulation of the oral mucosa. This can be because of repeated tobacco chewing, ill-fitted dentures, intentional or unintentional biting, sharp teeth, and so on. Because, clinically, fibromas resemble the features of other benign or reactive tumors, histological examination is required for the appropriate management of the same. Here, we describe a case of an irritative fibroma of the hard palate in a 61-year-old female. The patient had a history of betel nut and tobacco chewing for 30 years. The patient was evaluated and underwent complete excision for the same. The base of the lesion was cauterized to prevent recurrence.

## Introduction

An irritative fibroma is a benign, exophytic soft tissue lesion commonly found in the oral cavity. Rather than being a true tumor, it is a localized hyperplasia of fibrous connective tissue brought on by persistent irritation or trauma. Irritative fibroma is believed to have a complex etiology that includes chronic mechanical stress because of the presence of sharp teeth, abrasive foods, or poorly fitted dentures [1].

The most typical site is buccal mucosa, though the lesion may occur over the tongue and gingiva. Because of lesser chances of trauma or irritation, occurrence over the hard palate is quite uncommon [1].

Irritative fibromas account for 4.5% of all oral mucosal lesions [2]. Typically, they occur between the third to sixth decades of life. The incidence of irritative fibromas among the South Indian population was found to be 39.1% [3]. They are more in females, because of the contribution of hormonal effects [4].

There are very few reported cases of these lesions over the hard palate. Here, we are reporting a case of a 60-year-old female with a large irritative fibroma of the hard palate.

## Case presentation

A 61-year-old female presented to the Department of Otorhinolaryngology with the complaint of a persistent oral lesion that had been gradually increasing in size over the past two years. She had discomfort while chewing, particularly when eating hard foods. There was no history of trauma to the affected area or any associated symptoms, such as pain, bleeding, or burning sensation. The patient had a history of betel nut and tobacco chewing for the last 30 years. There was no significant medical history or any history of dental procedures.

**Figure 1:**
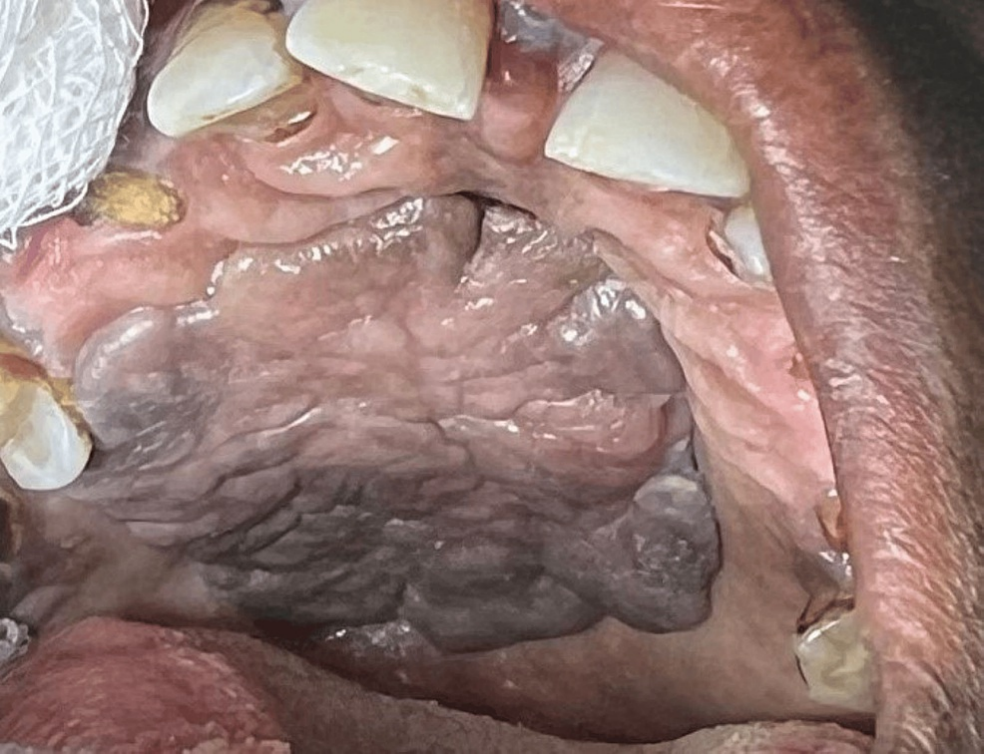
Hard palate: single, firm, greyish-pink mass seen covering almost the entire palate

On clinical examination, a single mass was seen arising from the right side of the hard palate. It was extending posteriorly and laterally crossing the midline. It was oval in shape, greyish-pink in color with irregular surface and margins, measuring 2.5 x 3.6 cm. On palpation, the mass was firm in consistency, non-tender, and mobile, and was attached by a stalk to the palatal mucosa. The rest of the palatal mucosa was normal in appearance. Dental condition was poor, and yellowish staining of teeth was present along with plaque formation (Figure 1). Neck examination was unremarkable with no enlargement of cervical lymph nodes.

**Figure 2:**
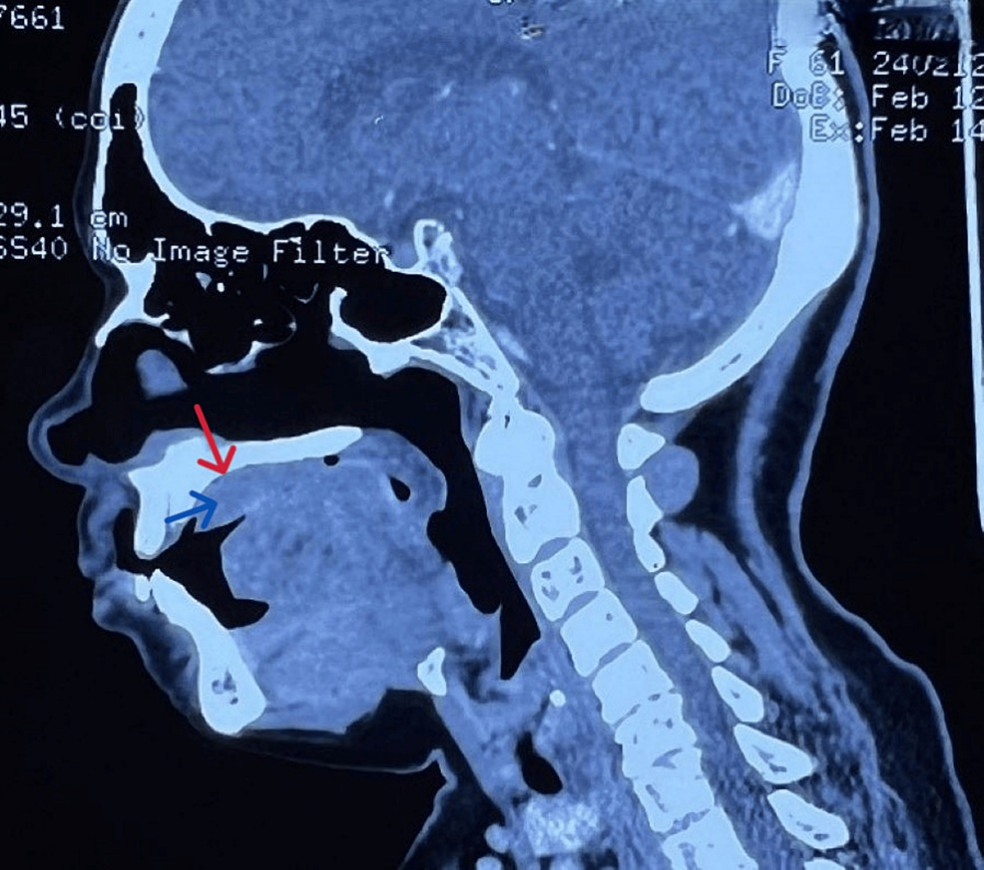
Contrast-enhanced CT of the face showing a soft tissue lesion covering the hard palate (marked by blue arrow), with an intact bony cortex above (marked by red arrow)

The patient underwent routine blood investigations, which were within normal limits. A contrast-enhanced CT of the face and neck was done. The scan revealed a soft tissue mass lesion of 3.5 x 3 cm overlying the right side of the hard palate extending to the left side by crossing the midline. The underlying bony cortex was well maintained. The fat planes on the posterosuperior aspect of mass were lost (Figure 2). A differential diagnosis of pyogenic granuloma, squamous papilloma, and an odontogenic tumor was considered. A biopsy sample was taken under local anesthesia and sent for examination to rule out the above possibilities.

**Figure 3:**
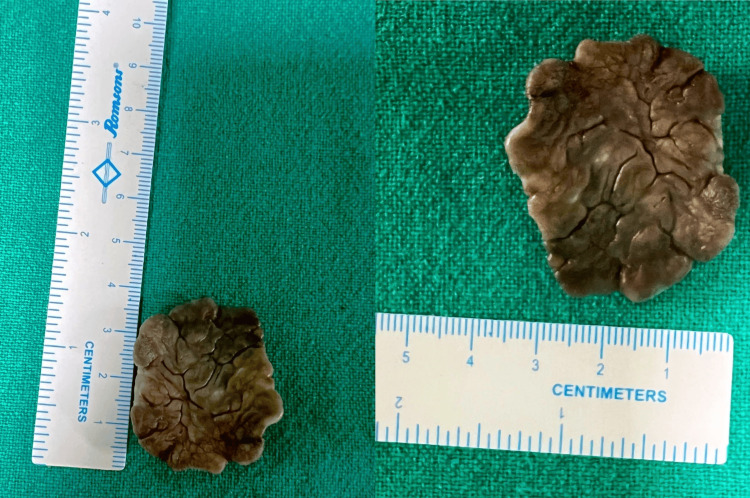
The lesion was excised completely, measuring 2.5 x 3.7 cm

Furthermore, the patient underwent a biopsy of the mass, which was suggestive of inflammatory papillary hyperplasia. A complete excision of the mass was done under general anesthesia (Figure 3).

**Figure 4:**
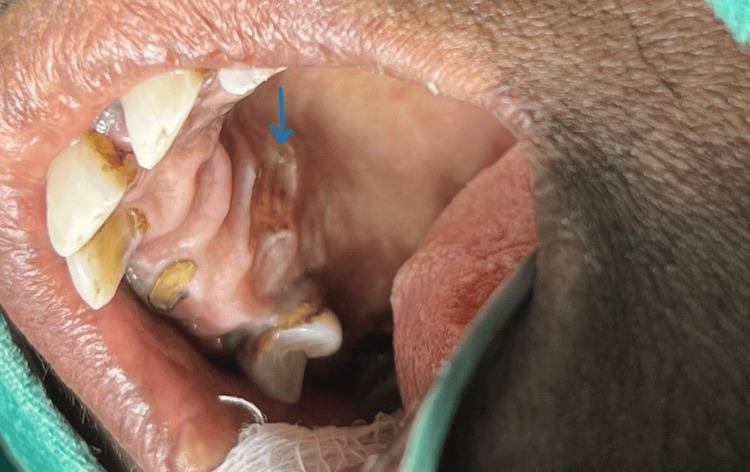
Right side hard palate-arrow showing a cauterized postoperated site

 The stalk was removed, and the base of the lesion was cauterized (Figure 4).

There was no bony defect in the palate postoperatively. The excised sample was sent for histopathological examination. No postoperative complications were seen.

**Figure 5:**
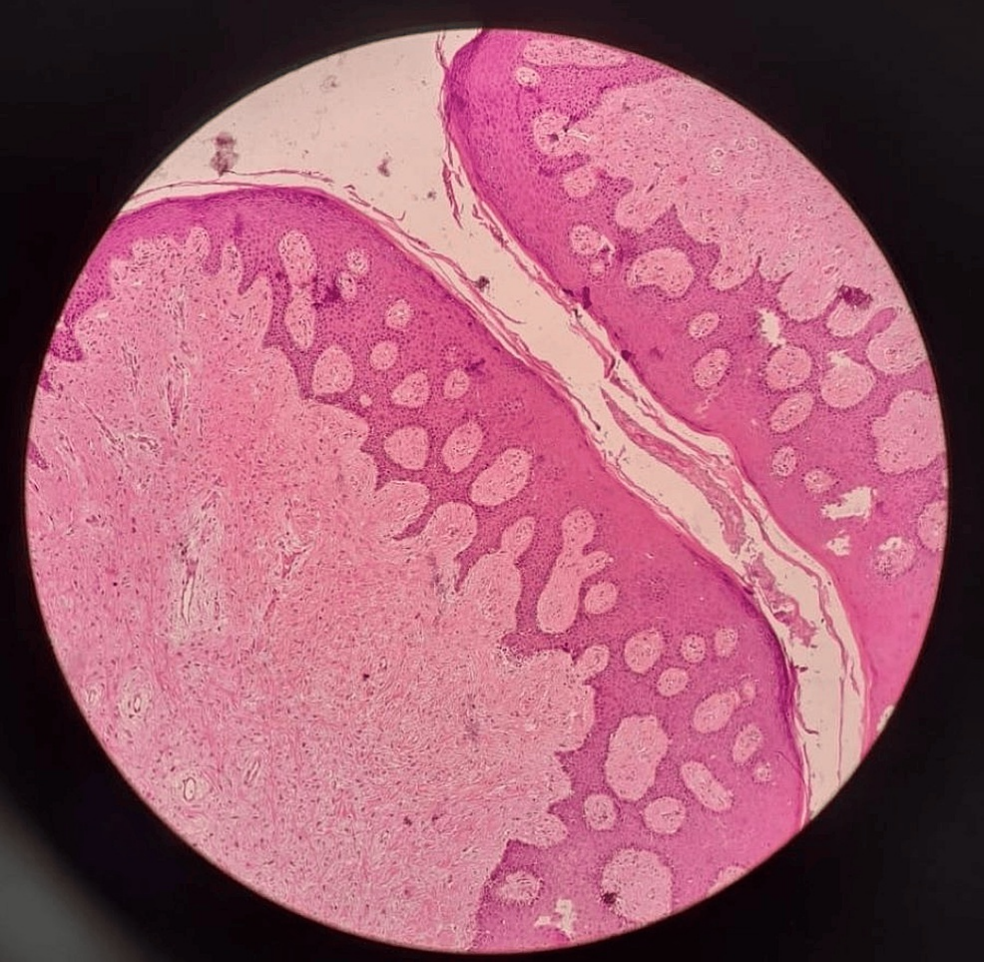
Histological section slide (10x magnification) Staining used: hematoxylin and eosin (H&E). The section shows a surface lined by stratified squamous epithelium, with the bulk of the lesion composed of dense, fibrous connective tissue, with varying degrees of collagen. There is a presence of chronic inflammatory cells, predominantly lymphocytes.

The final histopathological examination revealed fibro-collagenous connective tissue with collagen bundles containing fibroblasts. There was a presence of overlying squamous epithelium showing keratin collection. There was no definite evidence of dysplasia/malignancy (Figure 5).

The patient currently is on regular follow-up and doing well, with no signs of recurrence.

## Discussion

Although irritative fibromas are benign, they can lead to discomfort and interfere with oral function if left untreated. In a study, 283 irritative fibromas were recorded of 791 benign lesions of the oral cavity [4]. Among patients with 171 fibrous growths of the oral cavity, a higher occurrence of these lesions in females was reported. Out of 171 lesions, 45 lesions were reported to occur over the palate [4].

Fibromas usually affect the buccal mucosa because of local trauma and irritation. The hard palate is a rare site for occurrence. Various etiological factors lead to the alteration of oral mucosa. Betel chewing leads to persistent irritation of mucosa along with low-grade trauma to the palate by the upward movement of the tongue. Large fibromas are a result of low-grade injury to the palate and ongoing irritation allowing room for an asymptomatic tumor [2].

In terms of clinical presentation, they typically take the form of an exophytic nodule with solid to firm consistency, with a smooth, pink to flesh-colored surface. The nodule usually has well-defined margins.

The growth of these fibromas is slow and seldom exceeds 1.5 cm in diameter [5]. In our case, the size of the lesion was greater than in the reported cases. As the patient has noticed a small nodule-like lesion over the palate previously, it can be assumed that the small initial fibroma attained its current size because of ongoing irritation and trauma caused by betel nut and tobacco chewing.

Histologically, an irritative fibroma appears as a mass composed of fibrous connective tissue with collagen bundles. Because of chronic irritation, the epidermis may showcase hyperplasia or hyperkeratosis. In the connective tissue, there is a presence of dense collagen fibers and localized hyperplasia of mature fibroblasts [5].

A large palatal fibroma should be distinguished from other palatal swellings, both clinically and histologically. A differential diagnosis of pyogenic granuloma, necrotizing sialometaplasia, squamous papilloma, granular cell tumor, or odontogenic tumor should be considered [3], for differentiation from the aforementioned, appropriate radiological scans are required, especially to note any involvement or erosion of bone. Histopathological examination is crucial to distinguish malignant lesions leading to alteration of surgical modality.

The recommended course of treatment for an irritative fibroma is complete excision. Additional treatments such as cryosurgery or intralesional steroid injection may be explored, provided a conclusive histopathological examination [6].

Rarely, recurrence can develop from repeated trauma or irritation at the same site [2]. For prevention of recurrence, local causes of irritation such as tobacco chewing or betel nut chewing should be eliminated by the patient. Any ill-fitted dentures should be corrected and used after the complete healing of the surgical site. In our case, the patient was counseled to avoid betel nut chewing postoperatively. 

Incompletely excised lesions have had a high growth potential of 8%-20%, long-term surgical follow-up is crucial [2]. An adequate follow-up period is essential to monitor for recurrence and ensure complete healing. Typically, a follow-up period of six months postsurgical excision is recommended [1].

## Conclusions

An irritation fibroma of the hard palate is a rare clinical condition. Irritative fibromas are largely asymptomatic with no known malignant transformation potential. The chance of recurrence after complete excision is rare. Although benign, a complete surgical excision and a complete histological examination are warranted for diagnosis and differentiation from other neoplasms. A long-term follow-up is required in postoperative patients to prevent recurrence. Furthermore, local causes leading to this condition should be identified and treated/removed to prevent recurrence.
